# Multiplex Editing of *OsMads26*, *OsBsr-d1*, *OsELF3-2* and *OsERF922* with CRISPR/Cas9 Confers Enhanced Resistance to Pathogens and Abiotic Stresses and Boosts Grain Yield in Rice (*Oryza sativa*)

**DOI:** 10.3390/ijms27020781

**Published:** 2026-01-13

**Authors:** Hailing Luo, Hengwei Zou, Shengli Lin, Jiali Liu, Geng Zhou, Lijun Gao, Jieyi Huang, Jiaxuan Li, Ju Gao, Chonglie Ma

**Affiliations:** Guangxi Crop Genetic Improvement and Biotechnology Laboratory, Guangxi Academy of Agricultural Sciences, Nanning 530001, China; luohailing@gxaas.net (H.L.);

**Keywords:** rice, multiplex editing, resistance to pathogens, abiotic stress, grain yield

## Abstract

Rice (*Oryza sativa*) is one of the world’s major staple foods. However, stable rice production is constrained by various biotic and abiotic and stresses. Breeding and cultivation of rice varieties with resistance to multiple pathogens and environmental stresses is the most effective strategy to mitigate the adverse effect of pathogen attacks and abiotic stresses. Recently, researchers have focused on the exploitation of CRISPR/Cas9 technology to manipulate some negative defense-regulator genes to generate rice varieties with broad-spectrum resistance against rice pathogens. In this study, four negative regulator genes of rice blast, *OsMads26, OsBsr-1*, *OsELF3-2* and *OsERF922*, were selected as CRISPR/Cas9 targets. By simultaneously knocking out all four genes via CRISPR/Cas9 technology, we created three *mads26/bsr-1*/*elf3-2*/*erf922* quadruple knockout mutants. Our results demonstrated that all quadruple mutants exhibited much higher resistance not only to rice blast and bacterial blight but also to drought and salt stresses than the wildtype. Interestingly, grain yield of all three quadruple mutants was also drastically increased by 17.35% to 21.95%. Therefore, this study provides a novel strategy to rapidly improve rice varieties with broad-spectrum resistance to pathogens, elevated tolerance to abiotic stresses and enhanced yield potential.

## 1. Introduction

Rice blast caused by fungal pathogen *Magnaporthe oryzae* is one of the most destructive diseases of rice and seriously affects its yield. It is estimated that 10–30% of rice yield is lost annually due to rice blast [1]. Bacterial blight, caused by *Xanthomonas oryzae* pv. *Oryzae* (*Xoo*), is also a globally devastating rice disease. To deal with these pathogens, rice has evolved complex and sophisticated defense-responsive signaling pathways [2]. In the past few decades, considerable effort has been devoted to deciphering the molecular mechanisms underlying how rice promptly responds to pathogen attacks and effectively mitigates the adverse effects of diseases on rice growth and development, resulting in the identification of a large number of key regulatory genes that confer resistance to *M. oryzae* and *Xoo*. Among these cloned genes/alleles, blast resistance genes/alleles *Pi1* [3], *Pi2/Pi9/Pigm* [4] and *Pita* [5] exhibit durable and broad-spectrum resistance to *M. oryzae* and have been widely applied in rice blast resistance breeding. Bacterial blight resistance genes *xa5* [6], *Xa7* [7] and *Xa23* [8] also greatly contribute to current rice breeding of broad-spectrum resistance to *X. oryzae*. However, rice breeding of blast and bacterial blight resistance still faces challenges because only a limited number of major resistance genes/alleles or QTLs (quantitative trait loci) are currently available, and pyramiding multiple minor resistance genes into a variety may take a long time to achieve. Identification and creation of novel resistant genes/alleles and efficient methods to rapidly pyramid multiple resistant genes into a variety are still in high demand.

The discovery and application of CRISP/Cas9-mediated genome editing has led to a new era of plant breeding by providing researchers precise and efficient tools to modify crop genomes [9]. Using this method to manipulate plant immune circuits and develop crop varieties with broad-spectrum resistance to multiple pathogens or multiple isolates of a specific pathogen has become feasible [10]. Two recent reports highlight the significance of this technology. Wang et al. [11] have created a novel resistant *Xa23* locus with broad-spectrum and durable resistance to various isolates of *Xoo* and *Xanthomonas oryzae* pv. *Oryzicola* (*Xoc*) by installing 10 artificial EBEs (effector-binding elements) which were responsive to TALEs (transcriptional activation-like effectors) from various *Xoo* and *Xoc* strains into the susceptible *xa23^N46^* locus by genome editing. Sha et al. [12] have shown that a lesion mimic mutant (LMM), caused by a 29-base-pair deletion in a gene named RESISTANCE TO BLAST1 (RBL1), confers broad-spectrum disease resistance but with an approximately 20-fold reduction in yield. A 12bp in-frame mutant of RBL1 created by genome editing has demonstrated broad-spectrum resistant to *M. oryzae*, *Xoo*, *Xoc* and rice false smut *Ustilaginoidea virens* [12]. Using CRISPR/Cas9 technology to manipulate some negative regulators of the rice blast and bacterial resistance genes such as *Pi21* [13], *Bsr-d1* [14], *ERF922* [15] and *Xa5* [6] to enhance disease resistance has also become a hotspot recently. Using CRISPR/Cas9 editing technology to rapidly knockout a single negative defense-regulator gene *Bsr-d1* [16] or *ERF922* [17] enhanced resistance to rice blast. Disrupting the expression of three known broad-spectrum blast-resistant genes, *Bsr-d1*, *Pi21* and *ERF922* significantly improved the rice blast resistance of an elite two-line sterile line Longke638S [18]. Loss-of-function mutants of two S (susceptible) genes *Pi21* and *Bsr-d1* increased resistance to *M. oryzae*, and a knockout mutant of the S gene *Xa5* showed elevated resistance to *Xanthomonas oryzae* pv. *oryzae* (*Xoo*). Remarkably, the mutants of all three *S* genes had significantly improved resistance to both *M. oryzae* and *Xoo* [19]. Through targeted editing of two genes *Pi21* and *OsSULTR3;6*, the resistance to rice blast *M. oryzae* and *Xoc* (*Xanthomonas oryzae* pv. *Oryzicola*, the causal pathogen of bacterial leaf streak) was improved [20]. Most importantly, improved disease resistance does not compromise grain yield in all these cases, demonstrating that broad-spectrum resistance of rice blast and bacterial blight is achievable through CRISPR/Cas9 technology.

Rice improvement has continuously aimed to achieve higher yields and more substantial tolerance of various pathogens and abiotic stresses. In this study, we aimed to install four broad-spectrum resistance genes, *mads26, bsr-1*, *elf3-2* and *erf22*, into a japonica inbred variety ZG616 via CRISPR/Cas9 technology to improve its rice blast and bacterial blight resistance. Besides *OsBsr-d1* and *OsERF922*, which have been proven to be able to elevate resistance against rice blast and bacterial blight upon knocking-out through genome editing [16,18,19], we added other two rice blast resistance genes, *OsMad26* and *OsELF3-2*, to our CRISPR/Case9 targets. *OsBsr-d1* encodes a C2H2 transcription factor, which enhances broad-spectrum disease resistance by coordinating the reduction in hydrogen peroxide degradation [14]. *OsERF922* encodes an AP2/ERF-type transcription factor that is rapidly and strongly induced by ABA and salt treatment and is also induced by the rice blast fungus. The OsERF922 protein is localized in the nucleus and specifically binds to the GCC-box sequence, acting as a transcriptional activator. *OsERF922* negatively regulates rice resistance to the rice blast fungus and tolerance to salt [15]. *OsMADS26* encodes a transcription factor, and its protein contains a MADS-box domain. It acts as an upstream regulatory factor of stress-related genes, serving as a central regulator in rice’s response to various stresses. It negatively regulates resistance to rice blast and bacterial leaf blight. It also negatively regulates drought tolerance. Downregulation of *OsMads26* expression by RNAi (RNA interference) boosted rice resistance against rice blast and bacterial blight and improved the rice tolerance to drought without a significant effect on plant development or growth [21]. *OsELF3-2* is one of the orthologs of Arabidopsis *ELF3*, which encodes a protein homologous to a nematode response protein and plays an important role in flowering and circadian control [22]. The rice genome encodes two *ELF3* orthologs (*OsELF3-1* and -*2*), which share 75% amino acid identity. Unlike *OsEFL3-1* knockout mutants, which showed delayed flowering, the knockout and knockdown mutants did not differ from the wildtype plants in heading date. Instead, the knockout and knockdown mutants of *OsELF3-2* displayed a substantial increase in resistance to rice blast, suggesting that *OsELF3-2* negatively regulates resistance against *M. oryzae* [23]. In addition, *OsERF922* negatively modulates salt tolerance as well [15]. By simultaneously knocking out these four negative regulators of rice blast resistance with CRISPR/Cas9, we expected that the quadruple mutants of ZG616 would gain higher resistance against rice blast, bacterial blight and drought stresses. Indeed, three *mads26/bsr-1*/*elf3-2*/*erf922* quadruple knockout mutants we obtained through CRISPR/Cas9 technology displayed elevated resistance to not only rice blast and bacterial blight but also drought and salt stresses. Interestingly, grain yields of the quadruple mutants were drastically increased compared to those of the wildtype. This work provides a novel strategy to simultaneously improve rice’s defense against pathogens, tolerance to environment stresses and grain yield potential.

## 2. Results

### 2.1. Generation of Knockout Mutants of OsMads26, OsBsr-d1, OsELF3-2 and OsERF922

**Figure 1:**
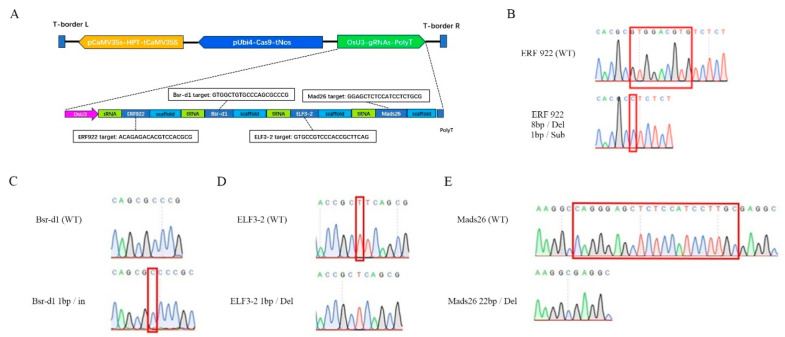
Schematic diagram of the T-DNA region of CRISPR/Cas9 editing vector pZZ636 (**A**) and representative sequencing confirmation of edited *OMad26*, *OsBsr-d1*, *OsELF3-2* and *OsERF922* genes (**B**–**E**). pCaMV35S, CaMV35S promoter; tCaMV35S, CaMV35S transcription terminator; pUbi4, sugarcane ubiquitin 4 promoter; tNos, Nos transcription terminator; OsU3, rice U3 promoter; Del, base deletion; In, base insertion; Sub, base substitution. The bases deleted, inserted or substituted are marked in red boxes on the sequencing chromatograms of wildtype genes and edited genes.

To knockout all the four rice blast resistance genes we selected in a single plant, a multiplex CRISPR/Cas9 editing vector pZZ636 was constructed by incorporating the four target gRNAs of *OsMads26*, *OsBsr-d1*, *OsELF3-2* and *OsERF922* genes into a single polycistronic gene (Figure 1A), a strategy described previously [24]. Transformation of pZZ636 into a japonica inbred variety ZG616 produced 161 T_0_ transformants. After transgene identification and screening of effective editing of each gene using methods described in detail in the Section 4, 141 T_0_ transformants with at least one gene being effectively edited were identified (Supplementary Table S1). Among these primary transformants, the editing efficiency fo*r OsMads26*, *OsBsrd1*, *OsELF3* and *OsERF922* was 89.4%, 83.1%, 88.0% and 72.5%, respectively. To avoid in-frame mutation, in which the number of bases deleted or inserted were multiples of three, the T_1_ plants of three mutants, #12, 72 and 115 (Supplementary Table S1), were chosen for further studies. First, about 200 T_1_ plants of each mutant were subjected to a GMO (genetically modified organism) test to eliminate transgene components. Second, PCR amplifications of each gene in each GMO-negative mutant and consequent sequencing of all PCR fragments were adopted to identify mutants with transgene-free, homozygous edited alleles for all the four genes. The representative sequencing results of each gene are shown in Figure 1B–E. Finally, three *mads26/bsr-1*/*elf3-2*/*erf922* quadruple mutant lines, named ZG616/CR3, ZG616/CR6 and ZG616/CR10, were obtained. All the insertions and deletions occurring in each gene would lead to a translational frame shift, suggesting that the function of all the four genes, *OsMads26*, *O*s*Bsrd1*, *OsELF3* and *O*s*ERF922*, would be disrupted in each of the three quadruple mutants ZG616/CR3, ZG616/CR6 and ZG616/CR10 (Table 1).

### 2.2. Knockout of OsMads26, OsBsr-d1, OsELF3-2 and OsERF922 Genes Confers Enhanced Resistance Against M. oryzae and Xoo

**Figure 2:**
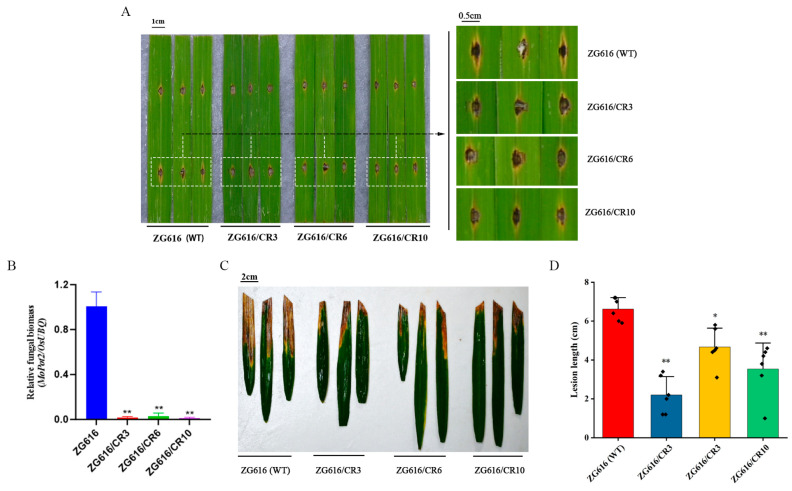
Knockout of *OsMads26*, *OsBsr-d1*, *OsElf3-2* and *OsERF922* elevates resistance to *M. oryzae* and *Xoo*. (**A**), Quadruple mutants were tested for resistance to *M. oryzae*. The picture was taken 5 days after inoculation. (**B**), Relative fungal biomass measured as the relative expression of *MoPot2* to that of *OsUbi 1*. (**C**), Quadruple mutants were tested for resistance to *X00*. (**D**), Lesion lengths (cm) caused by *Xoo.* *, *p* < 0.05; **, *p* < 0.01.

Previous studies demonstrate that *OsMads26*, *OsBsr-d1*, *OsElf3-2* and *OsERF922* are negative regulators of rice blast resistance, and downregulation or knockout of their expression leads to elevated blast resistance [14,16,17,23]. To investigate whether the quadruple mutants with all the four genes were simultaneously knocked out, detached leaves of three mutants and the wildtype were inoculated with the compatible *M. oryzae* isolate Couch 110-2. Five days after inoculation, quadruple mutants ZG616/CR3, ZG616/CR6 and ZG616/CR10 developed smaller disease lesions and accumulated less fungal biomass than the WT ZG616 (Figure 2A,B), implying that knockout of *O*s*Mads26*, *O*s*Bsr-d1*, *O*s*Elf3-2* and *O*s*ERF922* indeed elevated the resistance to rice blast as expected.

*OsMads26* negatively regulates resistance to bacterial blight [16]. We asked whether resistance to *Xanthomonas oryzae* pv. *Oryzae* (*Xoo*) was enhanced in quadruple mutants. At the booting stage, *Xoo* isolate PXO99A was inoculated to the fully expanded flag leaves of three mutants and the wildtype using the leaf-clipping method. Three weeks after inoculation, the leaf lesion length of bacterial blight was measured and analyzed. The results showed that the leaf lesion lengths caused by *Xoo* infection were drastically shorter for mutant plants than the wildtype (Figure 2C,D), suggesting that bacterial blight resistance was significantly elevated.

### 2.3. Knockout of OsMads26, OsBsr-d1, OsELF3-2 and OsERF922 Genes Elevates Drought and Salt Tolerance

**Figure 3:**
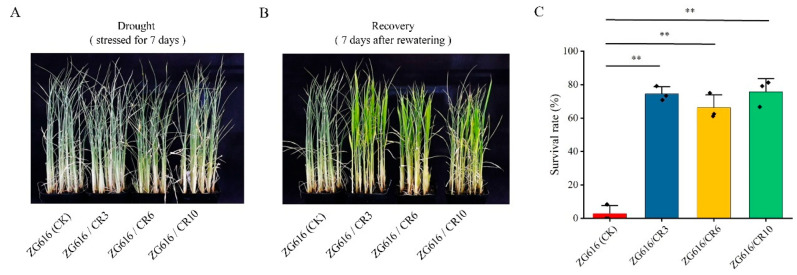
Quadruple mutants exhibit robust tolerance to drought stress. (**A**), Seedlings after stressed for 7 days. (**B**), Seedlings after 7 days of recovery. (**C**), The survival rates of quadruple mutants ZG616/CR3, ZG616/CR6, ZG616/CR10 and the wildtype ZG616. **, *p* < 0.01.

**Figure 4:**
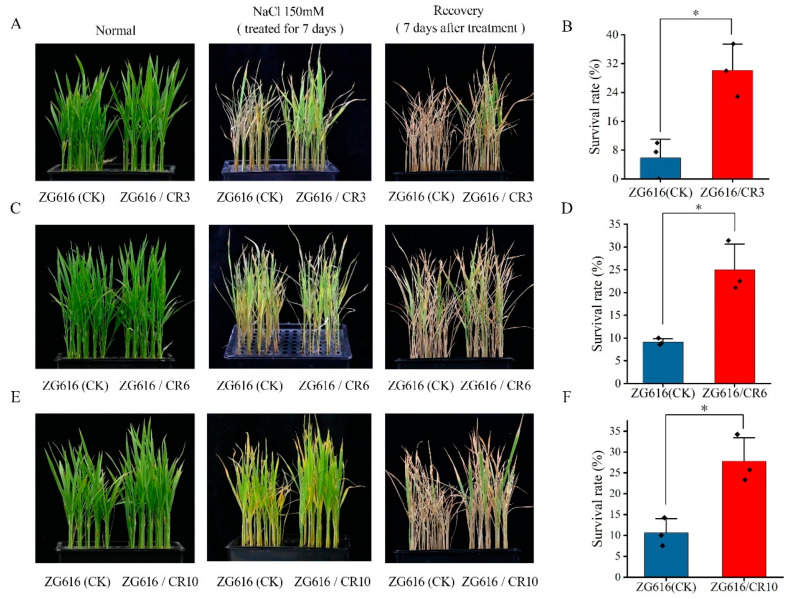
Salt tolerance tests of the quadruple mutants ZG616/CR3, ZG616/CR6, ZG616/CR10 and the wildtype ZG616. (**A**,**C**,**E**), Seedlings before salt stress (left panel), after salt stress for 7 days (middle panel) and after 7 days of recovery (right panel). (**B**,**D**,**F**), Survival rate of ZG616/CR3, ZG616/CR6 and ZG616/CR6 against the wildtype ZG616, respectively. *, *p* < 0.05.

Besides participating in regulation of rice blast resistance, *OsMads26* negatively modulates rice tolerance to drought and salt stresses as well [16]. In addition, *OsERF922* plays an important role in salt stress response [17]. Knocking down its expression via RNAi (RNA interference) results in better tolerance to salt stress [15]. We wondered whether the *mads26/bsr-1*/*elf3-2*/*erf922* quadruple mutants could tolerate drought and salt stresses better than the wildtype. In the drought tolerance test, watering of the seedlings of three mutants and the wildtype was withdrawn at the four-leaf stage. When curled leaves started to appear, seedlings were further stressed for 7 days followed by rewatering. We observed that the start time and the level of leaf curling did not show much difference between mutants and the wildtype (Figure 3A). However, the survival rate after 7 days of stress followed by 7 days of recovery displayed drastic difference between mutants and the wildtype (Figure 3B,C). The survival rates for mutants ZG616/CR3, ZG616/CR6 and ZG616/CR10 were 74.44%, 66.20% and 75.70%, respectively, whereas only 2.78% of the wildtype seedlings survived (Figure 3C). The difference between survival rates surely implied that drought tolerance of the quadruple mutants was dramatically improved compared to that of the wildtype. In the salt stress test, after the seedlings of three mutants and the wildtype grown in hydroponic culture medium reached the four-leaf stage, 150 mM NaCl was added to the hydroponic culture medium for salt stress treatment. Stress continued for 7 days followed by moving the seedlings back to normal hydroponic medium for recovery. After 7 days of salt stress, the seedlings of mutants showed less damage from high salt stress than the wildtype seedlings (Figure 4A,C,E) and had a substantially higher survival rate than the wildtype seedlings (Figure 4B,D,F). These results demonstrated that the quadruple knockout mutants had better salt tolerance than the wildtype.

### 2.4. Knockout of OsMads26, OsBsr-d1, OsELF3-2 and OsERF922 Genes Boosts Grain Yield Potential

**Figure 5:**
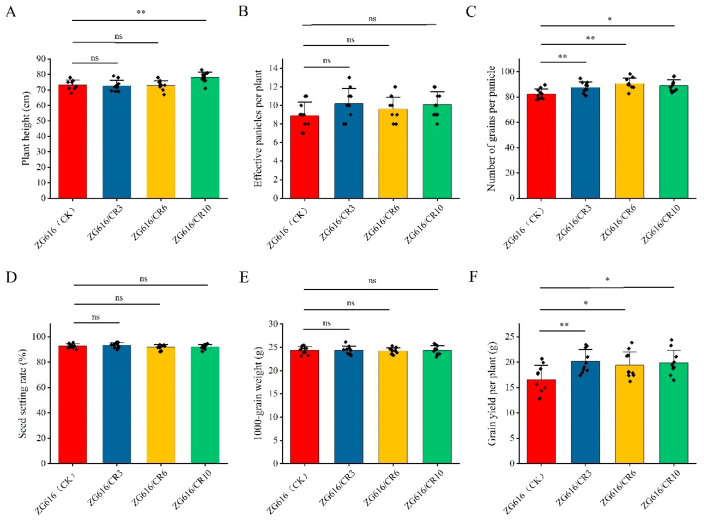
Major agronomic traits of quadruple mutants ZG616/CR3, ZG616/CR6, ZG616/CR10 and the wildtype ZG616. (**A**), Plant height (cm); (**B**), the number of effective panicles per plant; (**C**), the number of grains per panicles; (**D**), seed setting rate (%); (**E**), 1000-grain weight (g); (**F**), grain yield per plant (g). *, *p* < 0.05; **, *p* < 0.01 ns, not significant.

**Figure 6:**
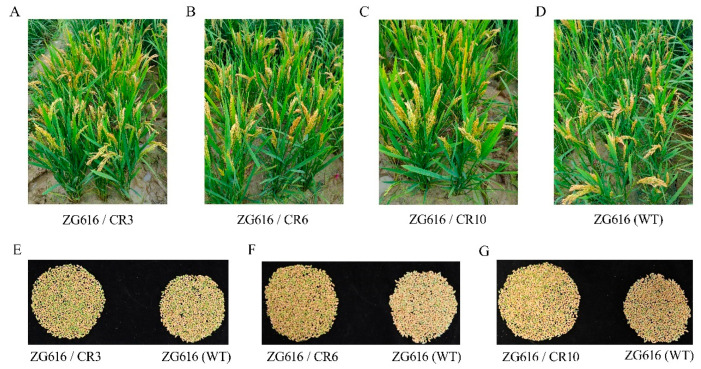
Quadruple mutants exhibit better grain yield potential. (**A**–**D**)**,** Mutant plants grown in the field. (**E**–**G**), Filled grains in total of a single representative mutant plant (left panel) and the wildtype (right panel).

The above results demonstrated that the *mads26/bsr-1*/*elf3-2*/*erf922* quadruple mutants were more resistant to rice blast and bacterial blight (Figure 2) and had elevated tolerance to drought (Figure 3) and salt (Figure 4). We further investigated whether increased resistance against rice blast, bacterial blight, drought and salt stresses could affect rice growth. At the mature stage, main agronomic traits, including the plant height, effective panicles per plant, number of grains per panicle, seed setting rate, thousand grain weight (TGW) and grain yield per plant were measured (Figure 5 and Figure 6). For plant height, only mutant line ZG616/CR10 exhibited a significant increase compared to the wildtype ZG616, whereas ZG616/CR3 and ZG616/CR6 did not display difference from the wildtype (Figure 5A). No significant difference was observed in the seed setting rate and TGW between wildtype ZG616 and the three quadruple mutants (Figure 5D,E). For effective panicles per plant, although statistical analysis showed that the effective panicles per plant between three mutants and wildtype was not significantly different, the average number of effective panicles per plant of three mutants was indeed higher than that of the wildtype (Figure 5B, Supplementary Figure S1). The number of effective panicles per plant was 10.20 ± 1.62, 9.60 ± 1.26 and 10.10 ± 1.37 for ZG616/CR3, ZG616/CR6 and ZG616/CR10, respectively, and 8.90 ± 1.45 for the wildtype ZG616, increasing by 14.60%, 7.87% and 13.48%, respectively. We also observed that three quadruple mutants appeared to have a higher panicle number per plant than the wildtype in the field (Figure 6A–D, Supplementary Figure S1). A significant increase in the number of grains per panicle occurred in mutants (Figure 5C). The average number of grains per plant increased by 6.67%, 10.04% and 8.13% for mutant ZG616/CR3, ZG616/CR6 and ZG616/CR10, respectively, compared to that of the wildtype. The slight increase in the number of effective panicles per plant and substantial increase in the number of grains per panicle, in turn, drastically increased the grain yield potential of the three quadruple mutants. The grain yield increased by 21.95%, 17.35% and 20.19% for ZG616/CR3, ZG616/CR6 and ZG616/CR10, respectively, compared to that of ZG616 (Figure 5F and Figure 6E–G), indicating that knockout of *OsMads26*, *OsBrs-d1*, Os*Elf3-2* and Os*Erf922* greatly boosted the grain yield potential of the quadruple mutants.

### 2.5. Multiple Biological Processes Involved in Stress Response Are Upregulated in Quadruple Mutants

**Figure 7:**
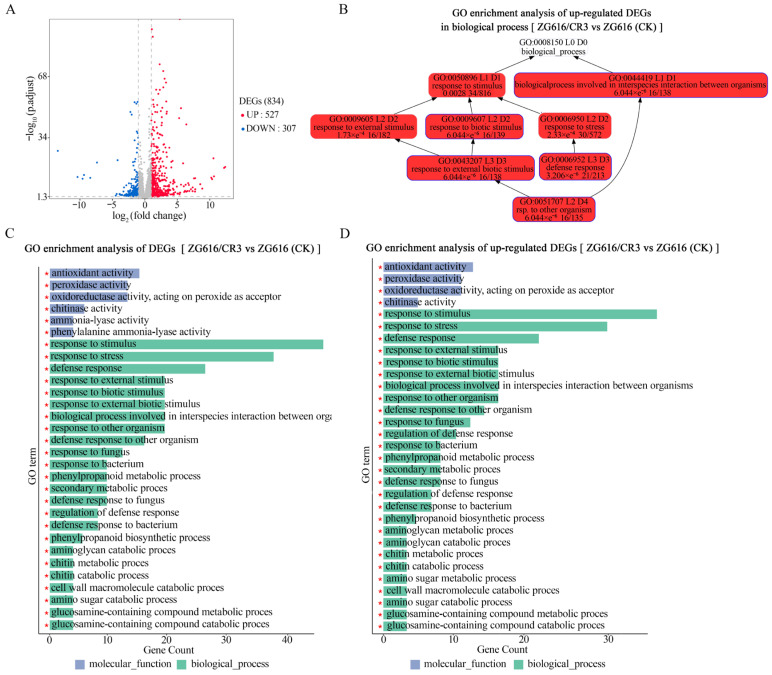
Analyses of the differentially expressed genes (DEGs) between quadruple mutant ZG616/CR3 and the wildtype ZG616 by RNA-seq. (**A**), The number of DEGs in total and the number of upregulated and downregulated DEGs. (**B**), DAG (Directed Acyclic Graph) of GO enrichment analysis of upregulated DEGs in the biological process; the darkness of the color represents the enrichment level, with red as the most significant level of enrichment. (**C**), Bar chart of GO enrichment analysis of total DEGs; only the top 30 enriched GO in terms of molecular function and biological process and the number of relevant genes are shown. (**D**), Bar chart of GO enrichment analysis of upregulated DEGs; only the top 30 enriched GO in terms of molecular function and biological process and the number of relevant genes are shown. The read * at the bottom of each bar means the GO term that the bar represents was significantly enriched.

The results presented above demonstrated that simultaneously knockout of *O*s*Mads26*, *O*s*Brs-d*1, *O*s*Elf3-2* and *O*s*Erf922* not only elevated resistance to rice blast, bacterial blight (Figure 2), drought (Figure 3) and salt (Figure 4) but also greatly boosted grain yield potential (Figure 5 and Figure 6). To gain more insights into the underlying molecular mechanism of these changes in important agronomic traits. RNA-seq of the transcriptomes of mutant ZG616/CR3 and wildtype ZG616 was carried out on leaves of 4-week-old seedlings grown under normal growing conditions. The results showed that 834 genes were differentially expressed, with 527 upregulated and 307 downregulated (Figure 7A) between ZG616/CR3 and ZG616. GO (gene ontology) analyses of the 834 DEGs (differentially expressed genes) revealed that genes involved in antioxidant activity, peroxidase activity and oxidoreductase activity (acting on peroxide as an acceptor) were significantly enriched (Figure 7C). The molecular functions of these genes are known to play pivotal roles in plant response to biotic and abiotic stress conditions [24]. Genes functioning in multiple biological processes related to disease resistance, including response to fugus and bacterium, defense response, biological processes involved in interspecies interaction between organisms, response to stress, etc., were highly enriched in the 834 DEGs (Figure 7B,C, Supplementary Figure S2). Most importantly, the 527 upregulated DEGs were also mainly clustered into molecular function and biological processes involved in stress response (Figure 7D), suggesting that major pathways linked to plant response to stresses could be constitutively pre-activated in the *mads26/bsr-1*/*elf3-2*/*erf922* quadruple mutants under non-stressful growing conditions. The pre-activated expression of stress response genes, in turn, might endow the quadruple mutants with higher resistance to rice blast, bacterial blight, drought and salt stresses as shown in Figure 2, Figure 3 and Figure 4.

## 3. Discussion

Rice blast and bacterial blight caused by pathogens *Magnaporthe oryzae* and *Xanthomonas oryzae* pv. *Oryzae* (*Xoo*), respectively, are two globally devasting rice diseases. Breeding and cultivation of rice varieties with broad-spectrum resistance are the most effective strategies to mitigate the adverse effect of pathogen attacks on rice production. Over the past few decades, researchers have identified plenty of broad-spectrum resistance genes/alleles/QTLs that give rice durable resistance against *M. oryzae* and *Xoo* [25]. Broad-spectrum resistance genes, including *Xa7* [7] and *Xa23* [8] for *Xoo* and *Pi2/Pi9/Pigm* [4] and *Pita* [5] for *M. oryzae*, have been widely used in rice breeding. However, introducing these genes or pyramiding them into rice varieties through crossing-based regular breeding strategy may take a long time to accomplish. With numerous toolboxes developed for the powerful CRISPR/Cas9 genome-editing technology [9], researchers have started to explore CRISPR/Cas9 techniques to rapidly introduce broad-spectrum resistance genes into elite rice varieties and parental lines of hybrid rice [11,12,16,17,18,19,20]. In this work, our major goal was to install broad-spectrum resistance to rice blast and bacterial blight into a japonica inbred ZG616 by multiplex editing of four genes, *O*s*Mads26*, *O*s*Brs-d*1, *O*s*Elf3-2* and *O*s*Erf922.* All the four genes negatively regulate rice blast resistance [14,15,21,23], and previous studies have demonstrated that knockout of *O*s*Bsr-d1* [16], *OsERF922* [17] or both [18,19] confers improved rice blast resistance. In addition, *OsMads26* participates in negative regulation of bacterial blight resistance [21]. The three quadruple knockout mutants obtained in this study indeed showed substantially improved resistance to both rice blast and bacterial blight (Figure 2). These results are consistent with previous studies [18,19], further confirming that editing multiple *S* (susceptibility) genes to rapidly introduce broad-spectrum resistance to rice varieties is a feasible option. However, only one isolate was tested for rice blast or bacterial blight resistance, and whether these quadruple mutants confer resistance to more isolates needs to be confirmed with further experiments.

Besides rice blast and bacterial blight diseases, drought and soil salinization stresses also greatly affect rice productivity and have adverse effects on rice growth and development [26,27]. For instance, drought affects approximately 42 million hectares of rice in Asia, with significant yield losses due to various degrees of drought stress at different rice growth stages [28,29]. We noticed that *OsMads26* negatively modulates drought stress resistance in addition to rice blast and bacterial blight resistance [21], and *OsERF922* is involved in negative regulation of salt tolerance [15]. With such functions of these two genes, all *mads26/bsr-1*/*elf3-2*/*erf922* quadruple knockout mutants displayed substantially improved tolerance to drought and salt stresses. They had much higher survival rates than the wildtype after drought and salt stress treatment (Figure 3 and Figure 4). These two beneficial traits were not observed in similar studies previously reported. This work indicates that exploitation of genes such as *OsMads26* and *OsERF922*, which are the center of both biotic and abiotic defense responses, may represent a new strategy to develop rice varieties with better disease resistance and environmental stress tolerance.

High yield and broad-spectrum resistance are two most important goals in crop breeding. Crop plants must quickly initiate an immune response when a pathogen invades to prevent the spread of infection. However, excessive or sustained activation of immune responses can inhibit plant growth and development, leading to a decrease in yield, a phenomenon known as the “growth–defense tradeoff”. Such a tradeoff is extremely common for lesion mimic mutant (LMM) genes. *lmm* mutants exhibit spontaneous, disease-like lesions without pathogen attack [30]. For example, *lmm* mutants *spl11* [31], *spl28* [32] and the aforementioned *rbl1* [12] confer broad-spectrum resistance to rice blast and bacterial blight but display severe growth and development defects and yield loss. There are some exemptions from the tradeoff. For instance, rice varieties with *bsr-d1* [14], *pi21* [13] or *Pigm* [4] gene are resistant to multiple *M. oryzae* isolates without yield penalty. Another excellent example is the well-known elite *IPA1* gene, which confers enhanced yield immunity [33]. Even more interesting is that *IPA1* also enhances tolerance to drought and cold stresses [34,35], implying that it bypasses the growth–defense tradeoff. The *mads26/bsr-d1/elf3-2/erf922* quadruple mutants created in this work not only improved resistance to pathogens and environmental stresses (Figure 2, Figure 3 and Figure 4) but also boosted grain yield (Figure 5 and Figure 6, Supplementary Figure S1), providing a new strategy to accomplish resistance improvement and yield increase at the same time. Although the molecular mechanism underlying these elevated beneficial traits remains to be elucidated, we speculate that the constitutive pre-activation of multiple defense response pathways detected by transcriptome profiling (Figure 7, Supplementary Figure S2) may contribute to the improvement of these traits. Though it is known that excessive or sustained activation of immune responses can be detrimental to plant growth and development, mild pre-activation of defense responses may be beneficial to growth, given the fact that rice plants are not always grown in ideal conditions. Of course, the yield measure was based only on a small sample (five plants for each line), which might compromise the accuracy of yield results. Carefully designed experiments are needed in the future to confirm whether the results reporting a yield increase of the *mads26/bsr-d1/elf3-2/erf922* quadruple mutants over the wildtype are scientifically sound.

In conclusion, by simultaneously knocking out *OsMads26*, *OsBrs-d1*, *OsElf3-2* and *OsErf922* in a japonica inbred variety ZG616 using CRISPR/Cas9 genome editing, we generated three quadruple mutants of ZG616. The mutants not only gained improved defense against pathogens and abiotic stresses but also increased grain yield potential. This work presents a novel strategy to rapidly improve rice varieties with better resistance to pathogens and abiotic stresses and achieve higher yield at the same time.

## 4. Methods and Materials

### 4.1. Plant Materials

The plants of ZG616, an inbred variety of japonica rice, and its three quadruple mutants produced in this study were grown in a greenhouse or growth chamber at 28 °C day/23 °C night with 12 h of light or in the field at our research station in Nanning (22°50′ N, 108°14′ E), Guangxi, PRC, with natural growing conditions.

### 4.2. sgRNA Design and Construction of Cas9 Vector

To select CRISPR/Cas9 targets, genomic sequences of 4 rice genes, *OsMads26*, *OsBsr-d1*, *OsELF3-2* and *ERF922*, were analyzed on the online toolkit [CRISPR-GE (Genome Editing)—Liu YG Lab]. One target with low off-target probability was chosen for each target gene (Supplementary Table S2). To express these 4 guide RNAs (gRNA), a strategy described previously [36] was adopted in this study. First, we designed a polycistronic gene by integrating all 4 gRNAs into the single polycistronic gene with each gRNA separated by a tRNA. OsU3 was added to the 5′ of the tRNA-gRNA array to drive the expression of the single polycistronic gene, and a polyT sequence was added to the 3′ of the tRNA-gRNA array for transcription termination. Second, the whole OsU3-polycistronic gene-polyT cassette with 2 additional restriction endonuclease sites PacI and AscI (New England Biolabs, Ipswich, MA, USA) at each end was synthesized by GenScript Biotech Co. (Nanjing, China) and subcloned into plasmid pUC57. Third, the editing vector pGGM636 was constructed by cutting out the entire synthesized gRNA expression cassette from plasmid pUC57-B4T with PacI and AscI restriction endonucleases and consequently ligating the recovered cassette DNA fragment into an editing vector pGGM216 digested with the PacI and AscI. The T-DNA (transfer DNA) region of the binary vector pZZ636 is shown in Figure 1A. In addition to the gRNA expression cassette, the pGGM T-DNA contained a CaMV35S-driven hygromycin phosphotransferase gene (for hygromycin resistance in plant cells) and sugarcane ubiquitin 4 promoter-driven *SpCas9* gene.

### 4.3. Plant Transformation

The editing vector pZZ636 was transferred into *Agrobacterium* tumefaciens strain EHA105 by electroporation and consequently delivered into ZG616 cells via *Agrobacterium*-mediated transformation as previously described [37].

### 4.4. Selection of Transgene-Free Homozygous Mutants

Genomic DNA of all hygromycin-resistant T_0_ plants was examined by PCR using the specific primers HPT-F and HPT-R. Subsequently, all PCR-positive plants were subjected to a sequence fragment length detective (FLD) assay. In the FLD assay, a DNA segment containing the up- and downstream of the editing target site of a gene with a length around 500 bp was amplified using a pair of gene-specific primers, a forward primer and a reverse primer of the GOI (gene of interest) with the forward primer modified with a 5′-FAM. The primer pairs of 4 target genes are listed in Supplementary Table S3. The lengths of the PCR products were detected by capillary electrophoresis using an Applied Biosystems 3730 sequencer. The DNA sequence changes of each gene in each T_0_ plant were documented and analyzed. The offspring of T_0_ plants were first subjected to GMO (genetically modified organism) testing using HPT specific primers HPT-F and HPT-R. GMO positive plants were eliminated. The negative plants were chosen for further PCR amplification of 4 target genes using gene specific primers (Supplementary Table S3), and the amplified PCR fragments were sequenced for DNA modification conformation. T_1_ plants with all 4 genes were successfully edited, and all 4 modified genes with homozygote status were selected for further phenotype analysis. To ensure there were no transgenic residues in selected homozygous mutants, we used 3 primer pairs (Supplementary Table S3) to amplify T-DNA components, including *CaMV35S* promoter, *HPT* and *Cas9*. Only homologous mutants with PCR product negative for all 3 primer pairs were selected for further analysis. All primers used in PCR amplifications are listed in Supplementary Table S3.

### 4.5. Magnaporthe Oryzae and Xoo Inoculation

For *M. oryzae* inoculation, when rice seedlings reached the four-leaf stage, the second leaf was detached from the plant. Two holes (3 cm apart) were produced on the middle of the second leaf with a hole punch. A suspension with a density of approximately 1~2 × 10^5^ spores/mL was prepared before inoculation by eluting 10–15 days-old *Magnaporthe oryzae* B.C. Couch 110-2 (isolated from Hunan province, China) using a 0.025% Tween 20 aqueous solution. Five to eight microliters (μL) of the previously prepared suspension were applied to the injured holes. The inoculated leaves were placed in a square Petri dish containing a 6-benzylaminopurine solution, and the Petri dishes were incubated at 28 °C. Three biological replicates were set for each mutant. The infection results were measured and photographed after 3–5 days. To analyze relative fungal biomass, DNA was extracted from rice leaves with lesions using the cetyltrimethyl ammonium bromide (CTAB) extraction method. DNA-based quantitative PCR (qPCR) was used to measure relative fungal biomass.

For *Xoo* inoculation, the leaf-clipping method was used. In brief, *Xanthomonas oryzae pv. Oryzae* isolate PXO99A was cultured in PPS medium overnight at 28 °C, and the bacteria were resuspended in sterile distilled water at a concentration of 0.6 (OD600). During the rice booting stage, three plants with 3–5 fully expanded flag leaves for each plant of the WTs and mutants were clipped approximately 2 cm from the leaf tip using a pair of scissors that had been dipped in the bacterial suspension. Plants were evaluated for lesion length.

### 4.6. Drought and Salt Resistance Assays

For salt resistance assays, healthy and plump seeds were selected, soaked in 55 °C warm water for 15–20 min, then soaked in cold water for 24 h. Once the radicle emerged, the geminated seeds were placed in a hydroponic tray (96 holes, 5mm diameter) filled with 1/2 Kimura B nutrient solution and grown in a growth chamber at 28 °C day/23 °C night with 14 h of light. When seedlings reached the four-leaf seedling stage, plants were subjected to salt stress by adding 150 mM NaCl to the nutrient solution. After seven days of salt stress treatment, plants were moved to the normal 1/2 Kimura B nutrient solution for recovery. After recovery for seven days, plant survival rate was investigated. Three biological replicates were set for WT and mutant plants.

For drought resistance assays, plants were germinated directly in soil and grown in the greenhouse. Each pot was filled with mixed soil (nutrient soil/vermiculite/humus = 1:3:3). Plants at the three- to four-leaf stage were subjected to 7–10 days of withheld water followed by 7 days of re-watering. Drought tolerance was evaluated by determining the percentage of plants that survived or continued to grow after the period of recovery. This experiment was performed using around 30 plants per line and repeated three times.

### 4.7. Measurement of Main Agronomic Traits

Wildtype (ZG616) and three mutant lines, ZG616/CR3, ZG616/CR6, ZG616/CR10, were planted in Nanning (Guangxi, PRC, 22°50′ N, 108°14′ E). Each line was planted in five rows with eight plants in each row. At the maturity stage, five plants were randomly selected to investigate the plant height, effective panicle number, panicle length, the number of grains per panicle and 1000-grain weight grain yield/plant. Then, the data were analyzed using Excel and IBM SPSS Statistics 20.

### 4.8. RNA-Seq and Bioinformatic Analysis

To obtain rice materials for RNA-seq, the leaves of 4-week-old seedlings were sampled. The RNA-seq was conducted by Gene Denovo Biotechnology Co. (Guangzhou, China) on an Illumina Novaseq6000 (San Diego, CA, USA). DESeq2 was used to identify differentially expressed genes (DEGs) with the following thresholds: false discovery rate (FDR) < 0.05 and fold change ≥ 2. Gene ontology (GO) and Kyoto Encyclopedia of Genes and Genomes (KEGG) analyses were carried out with previous methods [38].

## Figures and Tables

**Table 1:** Genotype of edited mutant lines.

Edited Gene	Mutant Line and Details of Each Edited Gene		
	ZJ616/CR3	ZJ616/CR6	ZJ616/CR10
*OsMads26*	−22	−7	−C
*OsBsr-d1*	+A	−26	+C
*OsELF3-2*	−T	−5	−4
*OsERF922*	+G	+G	−8, G→C

+, insertion; −, deletion; the letter or number following the symbol represents the exact base inserted/deleted or the number of bases inserted/deleted, respectively. G→C, base substitution.

## Data Availability

The original contributions presented in this study are included in the article/Supplementary Materials. Further inquiries can be directed to the corresponding authors.

## Supplementary Materials

The following supporting information can be downloaded at: https://www.mdpi.com/article/10.3390/ijms27020781/s1.
