# Supplementary material for: Multiplex Editing of OsMads26, OsBsr-d1, OsELF3-2 and OsERF922 with CRISPR/Cas9 Confers Enhanced Resistance to Pathogens and Abiotic Stresses and Boosts Grain Yield in Rice (Oryza sativa)
Source: Int J Mol Sci. 2026 Jan 13;27(2):781. doi: 10.3390/ijms27020781 (PMC12840674; doi:10.3390/ijms27020781)
Supplement: Supplementary file 1 [file ijms-27-00781-s001.zip › ijms-4074478-supplementary.pdf]

Table S1. Genotypes of T_0 transformants.

T_0 plant No.	MADS26	Bsrd1	ELF3	ERF922	T_0 plant No.	MADS26	Bsrd1	ELF3	ERF922
1	-14/+1	0	-4/-2	-1	36	-2/+1	-3/+3	-4/-1	-1/+1
2	-1	+1	-3/-1	0	37	0	-1	0	+1/-4
3	-18/+1	-1/+3	-4	+1	38	0	+1	0	0
4	+1	+1	+1	-22/+1	39	-2	0	-2/0	0
5	-3/+1	+2/+3	-3	-24/-12	40	-15/-5	+2	-2	-24/-23
6	-18/-2	0	-2/+1	+1	41	-2/+1	+3	-2	-3/+1
7	-3/-2	+2	-2	0	42	-12/+1	-1/+1	-4/-3	-23/+1
8	-2/-1	+2	-5/+1	+1	43	-3/+1	0	-1	+1
9	-2	+1/+2	-1	-25/-2	44	0	0	-1	-1
10	0	0	-1	-6/+1	45	0	0	0	+1/-5
11	-5/+1	0	-4/-2	0	46	-1/+1	+2	-2	0
12	-22/-5	+1/+3	-1	+1	47	-5/+1	0/+2	-4/-1	+1
13	-14/-1	+1/+3	-3	-1/+1	48	0	0	0	-1
14	-10/-2	+3	-1	-6/0	49	-2/+1	+2	-4/0	0
15	-2	+3	-3	-28/-8	50	-3/+1	+3	-6/-2	-1
16	-8/+1	+3	-4/+1	+1	51	-1	0/+2	0	0
17	-5/-1	+1	0	0	52	-4/+1	0	-3/0	-16/0
18	+1	+3	-2	+1	53	+1	+3	-3/-1	0
19	-3/+1	-7/+2	-2	-50/0	54	-5/+1	-13/+2	-4/-1	-15/0
20	-5/-1	+1/+3	0	0	55	-27/-4	+2	-2/-1	-6/0
21	-6/-4	0	-1	-8/0	56	-1	0	-18/-3	0
22	-3/-2	+2	-4	0	57	-2/-1	-6/+3	-3	-62
23	+1	+3	-4	-7	58	-2	0/+2	-4/-3	-25/0
24	-27/-5	+3	0	0	59	-14/-10	+2	-3/-1	-9/0
25	-2/-1	+1/+3	-1	-23/+1	60	-13/-3	-1/+2	-6/-2	-1
26	-14/-2	+3	-2/0	0	61	-12/-2	0/+2	-8/-1	0
27	-5/-2	+3	-2	0/+1	62	-11/-1	0/+2	-4/0	-25/0
28	+1	+3	0	-11/+1	63	0	-13/+2	0	0
29	-15/-8	+3	-2	-37/-25	64	0	0	-2	-1
30	-2/+1	+1	-51/	-24/-9	65	-5/+1	+2	-4/0	0
31	-2	+3	-1/+1	0	66	-18/-8	+2	-38/+1	-3/+1
32	-4	+3	-3/-1	-8	67	-3/+1	-13/+2	-4/-3	-22/-1
33	-4/-1	+2	-4/-2	-29/0	68	-5/-2	+3	-3/-1	-16/0
34	0	0/+2	-4/-1	-23/+1	69	+1	0/+3	-1	0
35	0	0	0	-13/+1	70	-7/-4	+2/+3	-4/-3	-1/0
71	-1	-20	-3	-6/0	107	-13/-11	+2	-2	-25
72	-7/+2	-26/+3	-5/+1	+1/0	108	-5/-2	+2/+3	-3/-1	0
73	-23/-6	+2	-1	0/+1	109	-3/-2	-2/+3	+1	-1/+1
74	-25/+1	+1/+2	-2/0	-1	110	-4	0	0	-3/+1
75	0	0	-1	0	111	-40/-2	+1/+3	-4/-1	0
76	-4/-3	0/+3	-3/-1	0	112	-2/+1	3	-13/-3	+1/0
77	-4/-2	+1/+3	-5	-6/0	113	-18/-2	0	0	-17/+2
78	-7/-4	0/+3	-9/-3	-64/-1	114	-2	0/+2	0	0
79	-3/-2	+3	-3	-5/0	115	-21/-1	+1	-3/-4	-8/0
80	-2/+1	+2/+3	-3	-35/0	116	-2	-5/+2	-6/-2	0
81	-1/+1	0/+2	-3/+1	-4/-1	117	-2/0	+2/+4	-4	-1/0

82	-3/-2	0/+3	-3/0	0	118	-2/+1	+3	-3/-1	0/+1
83	-7/+1	-9/0	-3	-1	119	-14/-3	-18/-15	-5/-2	0
84	-5/-1	0	-4/+1	+2	120	-5/-3	0	-1	0
85	-2/-1	-1/+3	-4/-3	-2/+1	121	-1	-6/0	-115/-3	-21/0
86	-4/-2	-2/+2	-5/-4	0	122	-1	+2	-3	-4/0
87	-10/-1	0	-1	0	123	-5/-1	0/+2	-3/-1	0
88	-4/-2	+3	-3	-1/0	124	0/+2	0	-3/-1	-1
89	-23/0	+1/+2	0	0	125	-9/-5	+3/+5	-7/-1	0
90	-3/-2	+3	-5	0/+51	126	-9/-5	+3/+5	-3/0	0
91	-2/+1	0/+2	0	+1/-1	127	-3/-1	-4/+2	+1	+1
92	-2/0	0	0	-1	128	-5/-3	+2/+3	-2	0
93	-2/+1	+2	-4/-2	-1/0	129	-1	+3	-3/0	-12/0
94	-2/-1	+2/+3	-3	0	130	-1	0	-1	-1
95	0	0	0	-3/+1	131	-1	0	-1	-2
96	-4/-1	-1	-2	0	132	-5	+2	-5/-3	-1
97	-11/-10	0/+3	-2	+2/0	133	-3	+3	-3	0/+1
98	-2/-1	+3	-3/-1	+1	134	-1	+3/+5	-3/-1	+1
99	-5/-1	-6/+3	-5/-4	-6/0	135	-3/-1	-7/+3	-3/-1	+1
100	-10/-3	0/+3	-3/0	0	136	-3/+2	+3	-2	+1
101	-1/+1	-3/+2	-3/-1	+2/-1	137	-1/+2	0/+5	-1/0	-9/+1
102	+1	-18/0	-3/0	0	138	0	0	+1	+1
103	-5/+1	0/+3	-3/-1	-3/+1	139	0	+2	+1	+2
104	-2/+1	+3	-17/-2	-25/0	140	-1	+3	-3/0	0
105	-1/+1	0/+2	-1	0	141	-26/-23	0/+2	-1/+1	-9
106	0	-1	-2	+5/-1	142	0/+2	-4/0	-4/-3	-5/+1

+, insertion -, deletion, the letter or number followed by represents the bases inserted/deleted or the number of bases inserted/deleted, 0, no base change. Highlighted with yellow-colored background is the T_0 transformants selected for screening quadruple mutants.

Table S2. Target sequence and PAM sequence (underlined) of target genes.

Gene	Target sequence and PAM
OsERF922	ACAGAGACACGTCCACGCG **<u>TGG</u>**
OsBsr-d1	GTGGCTGTGCCCAGCGCCCG **<u>CGG</u>**
OsELF3-2	GTGCCGTCCCACCGCTTCAG **<u>CGG</u>**
OsMads26	GGAGCTCTCCATCCTCTGCG **<u>AGG</u>**

Table S3. Primers used in this work.

Primer name	Primer Sequence
Mads26 F	GGAGCTATCGATCATCAAGC
Mads26 R	GGCTAGCTAACAACTGAAATGG
Bsr-d1 F	CAACGGGGACAGATATGACG
Bsr-d1 R	TTCGAGTCACCAGCGGATG
ELF3-2 F	ACCTGCGGCCTACGAATCGATCG
ELF3-2 R	TTGAATACGCACGCAACGGAAGG
ERF922 F	CACCTCTCTCGATCGTCTCAT
ERF922 R	CGCTCCCCTGCCTCC
Cas9-F	CAAGCAGTCCGGCAAGAC
CS9-R	GGACACCTGGGCCTTCTG

CaMV35S-F	CGACAGTGGTCCCAAAGA
CaMV35S-R	AAGACGTGGTTGGAACGTCTTC
HPT-F	GACATTGGGGAGTTTAGCGAGAG
HPT-R	ACGAGGTACCGGACTTCGC

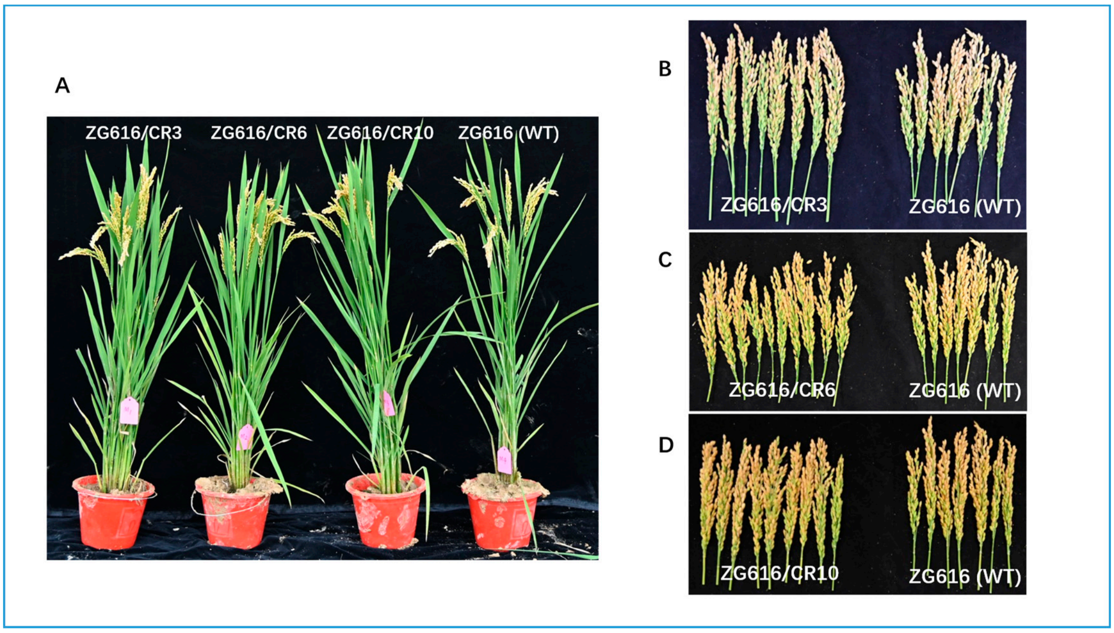

Figure S1. Photographs of representative plants of quadruple mutants and the wild type **(A)** at mature stage, and panicles from a single plants of quadruple mutants and the wild type **(B-D)**.

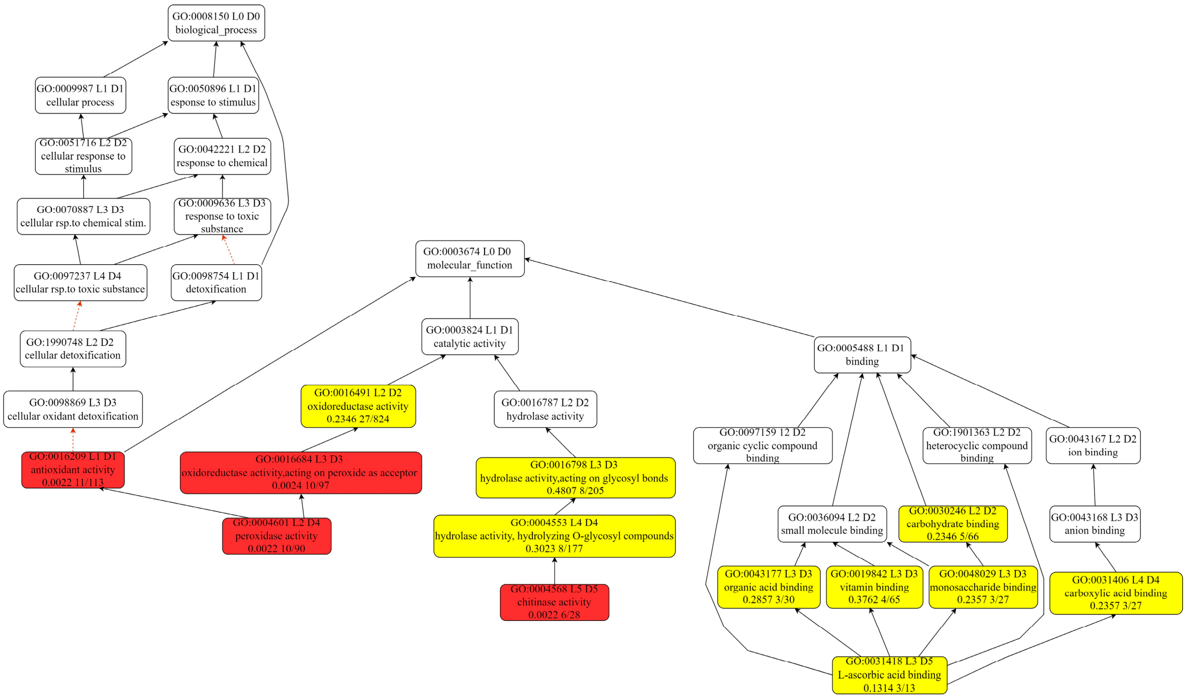

(A)

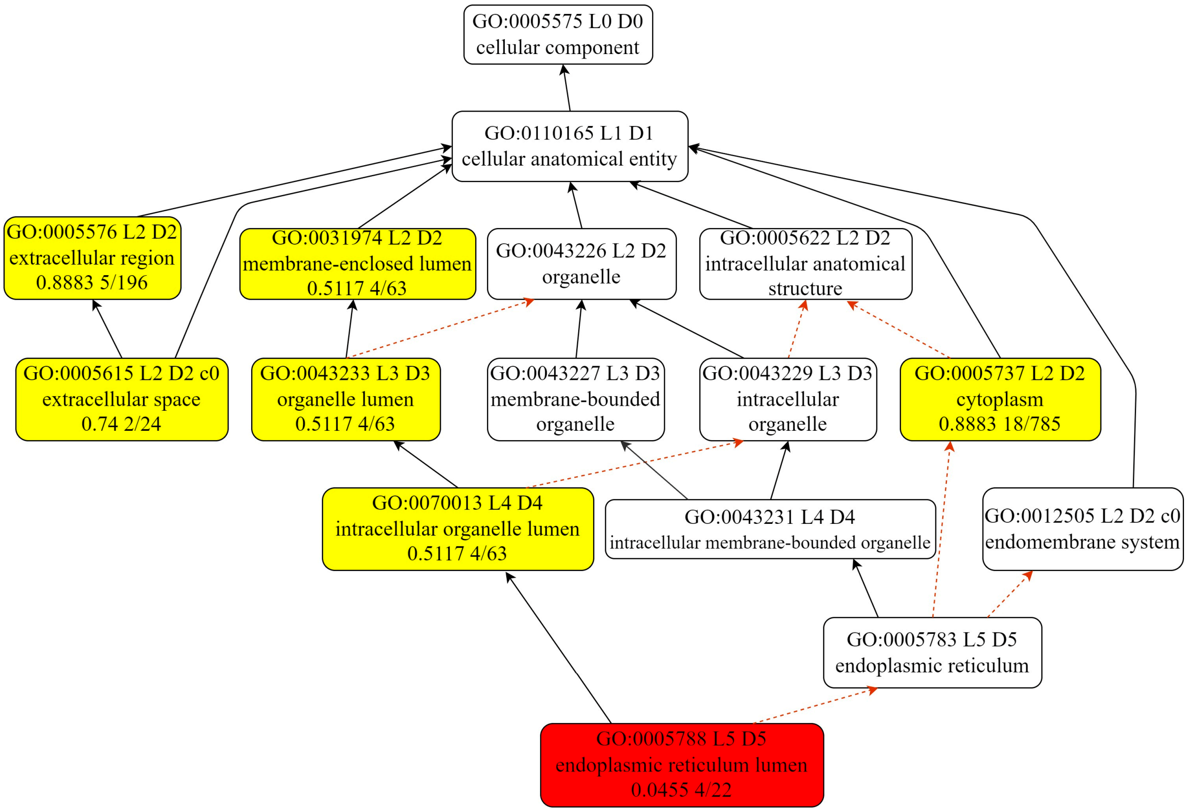

GO:0005575 L0 D0
cellular component
GO:0110165 L1 D1
cellular anatomical entity
GO:0005576 L2 D2
extracellular region
0.8883 5/196
GO:0031974 L2 D2
membrane-enclosed lumen
0.5117 4/63
GO:0043226 L2 D2
organelle
GO:0005622 L2 D2
intracellular anatomical
structure
GO:0005615 L2 D2 c0
extracellular space
0.74 2/24
GO:0043233 L3 D3
organelle lumen
0.5117 4/63
GO:0043227 L3 D3
membrane-bounded
organelle
GO:0043229 L3 D3
intracellular
organelle
GO:0005737 L2 D2
cytoplasm
0.8883 18/785
GO:0070013 L4 D4
intracellular organelle lumen
0.5117 4/63
GO:0043231 L4 D4
intracellular membrane-bounded organelle
GO:0012505 L2 D2 c0
endomembrane system
GO:0005783 L5 D5
endoplasmic reticulum
GO:0005788 L5 D5
endoplasmic reticulum lumen
0.0455 4/22

(B)

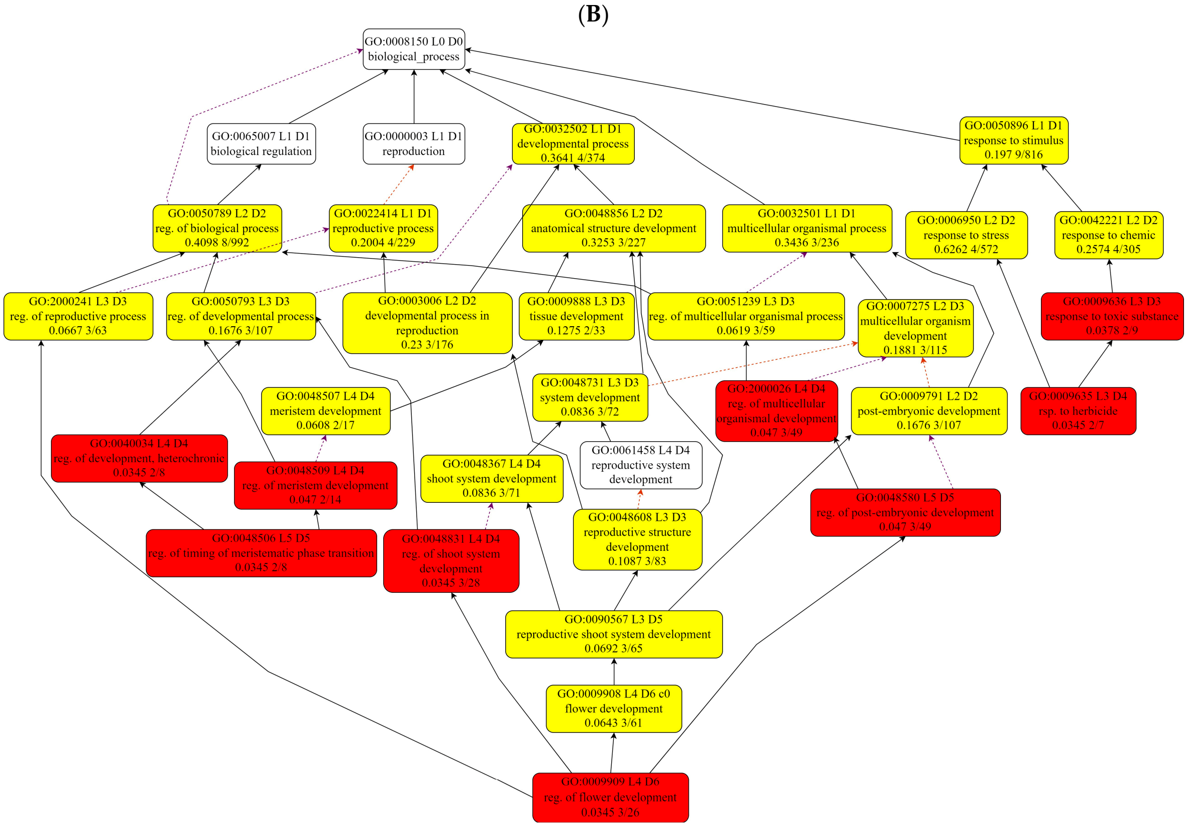

GO:0008150 L0 D0
biological_process
GO:0065007 L1 D1
biological regulation
GO:0000003 L1 D1
reproduction
GO:0032502 L1 D1
developmental process
0.3641 4/374
GO:0050896 L1 D1
response to stimulus
0.197 9/816
GO:0050789 L2 D2
reg. of biological process
0.4098 8/992
GO:0022414 L1 D1
reproductive process
0.2004 4/229
GO:0048856 L2 D2
anatomical structure development
0.3253 3/227
GO:0032501 L1 D1
multicellular organismal process
0.3436 3/236
GO:0006950 L2 D2
response to stress
0.6262 4/572
GO:0042221 L2 D2
response to chemic
0.2574 4/305
GO:2000241 L3 D3
reg. of reproductive process
0.0667 3/63
GO:0050793 L3 D3
reg. of developmental process
0.1676 3/107
GO:0003006 L2 D2
developmental process in
reproduction
0.23 3/176
GO:0009888 L3 D3
tissue development
0.1275 2/33
GO:0051239 L3 D3
reg. of multicellular organismal process
0.0619 3/59
GO:0007275 L2 D3
multicellular organism
development
0.1881 3/115
GO:0009636 L3 D3
response to toxic substance
0.0378 2/9
GO:0048507 L4 D4
meristem development
0.0608 2/17
GO:0048731 L3 D3
system development
0.0836 3/72
GO:2000026 L4 D4
reg. of multicellular
organismal development
0.047 3/49
GO:0009791 L2 D2
post-embryonic development
0.1676 3/107
GO:0009635 L3 D4
rsp. to herbicide
0.0345 2/7
GO:0040034 L4 D4
reg. of development, heterochronic
0.0345 2/8
GO:0048509 L4 D4
reg. of meristem development
0.047 2/14
GO:0048367 L4 D4
shoot system development
0.0836 3/71
GO:0061458 L4 D4
reproductive system
development
GO:0048580 L5 D5
reg. of post-embryonic development
0.047 3/49
GO:0048506 L5 D5
reg. of timing of meristematic phase transition
0.0345 2/8
GO:0048831 L4 D4
reg. of shoot system
development
0.0345 3/28
GO:0048608 L3 D3
reproductive structure
development
0.1087 3/83
GO:0090567 L3 D5
reproductive shoot system development
0.0692 3/65
GO:0009908 L4 D6 c0
flower development
0.0643 3/61
GO:0009909 L4 D6
reg. of flower development
0.0345 3/26

(C)

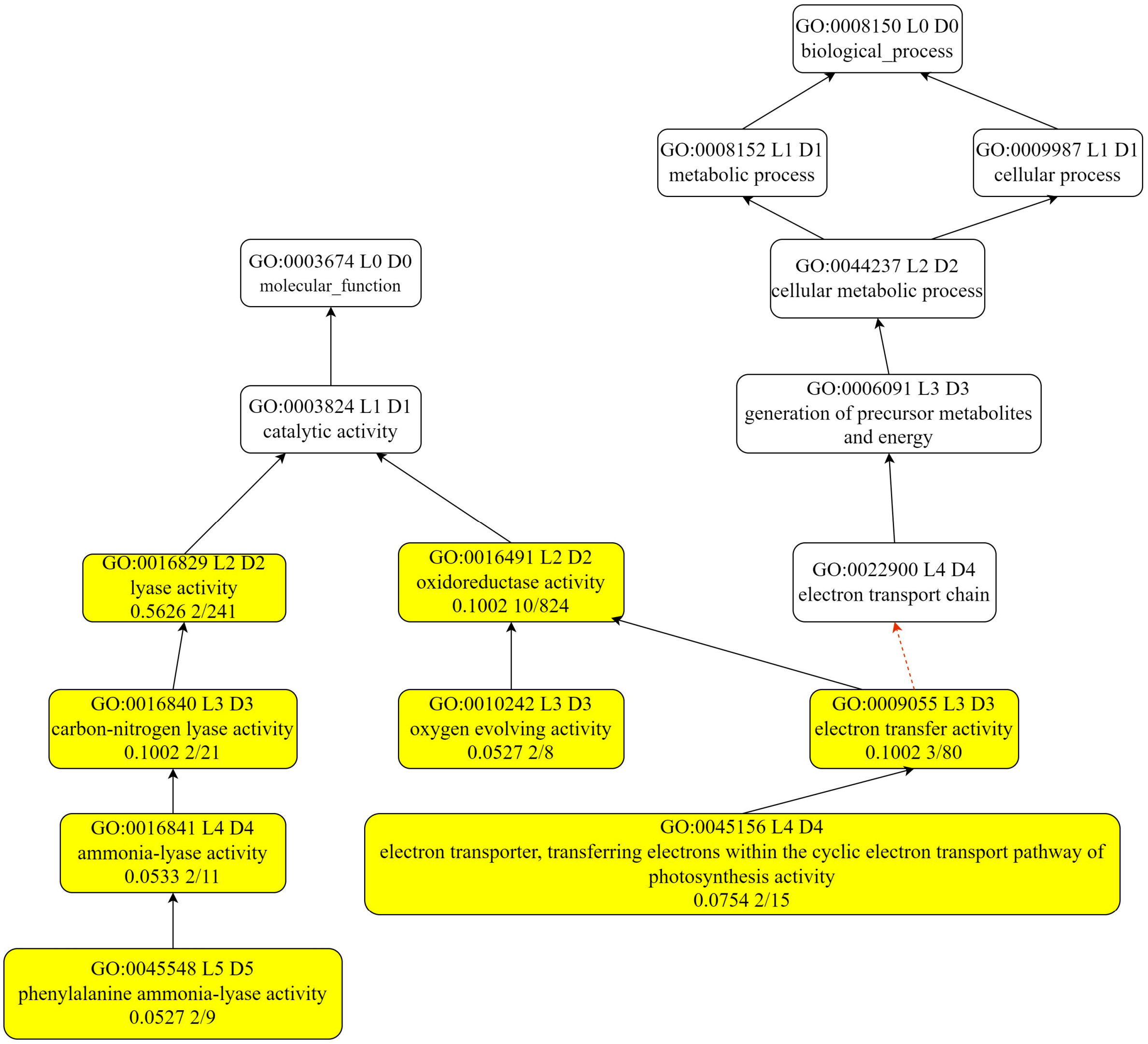

(**D**)

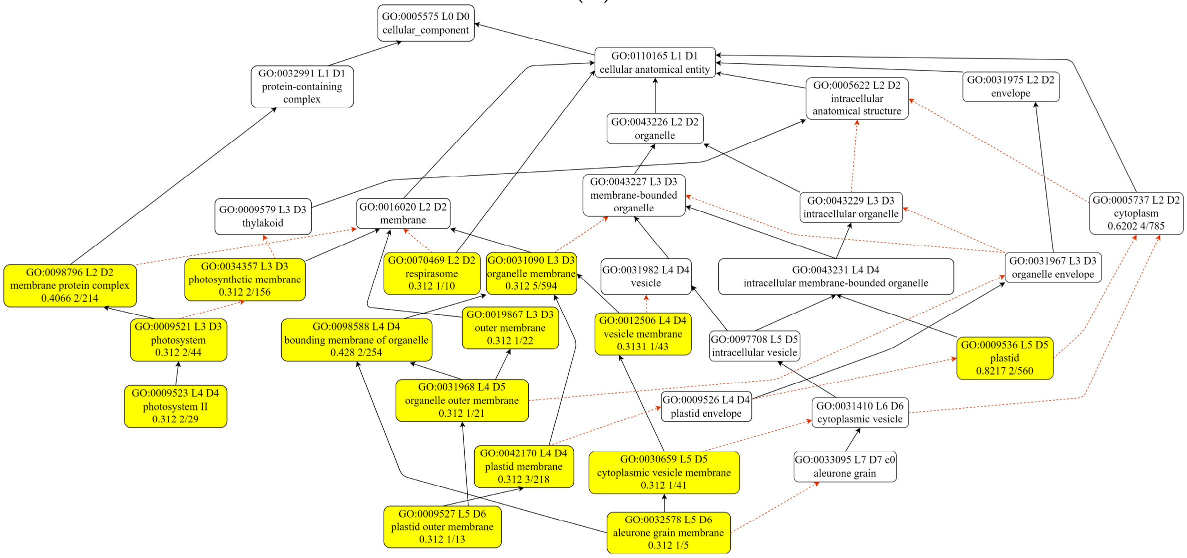

(**E**)

Figure S2. Analyses of the differentially expressed genes (DEGs) between quadruple mutant ZG616/CR3 and the wild type ZG616 by RNA-seq. DAG (Directed Acyclic Graph) of Go enrichment analysis of up-regulated DEGs in molecular function **(A)** and cellular component **(B)**. DAG (Directed Acyclic Graph) of Go enrichment analysis of down-regulated DEGs in biological process **(C)**, molecular function **(D)** and

cellular component **(E)**. The darkness level of color represents the enrichment level with red color as the most significant enrichment.
